# Developing a feasible fast-track testing method for developmental neurotoxicity studies: alternative model for risk assessment of micro- and nanoplastics

**DOI:** 10.3389/ftox.2025.1567225

**Published:** 2025-04-15

**Authors:** Xian Wu, TinChung Leung, Dereje D. Jima, Majemite Iyangbe, John Bang

**Affiliations:** ^1^ Department of Pharmacology and Toxicology, Brody School of Medicine, East Carolina University, Greenville, NC, United States; ^2^ The Julius L. Chambers Biomedical and Biotechnology Research Institute, North Carolina Central University, Durham, NC, United States; ^3^ Department of Biological and Biomedical Sciences, College of Health and Sciences, North Carolina Central University, Durham, NC, United States; ^4^ Center for Human Health and Environments, North Carolina State University, Raleigh, NC, United States; ^5^ Bioinformatics Research Center, North Carolina State University, Raleigh, NC, United States; ^6^ Intergrated Bioscience, Ph.D. Program, North Carolina Central University, Durham, NC, United States; ^7^ Department of Environmental, Earth, and Geospatial Sciences, College of Health and Sciences, North Carolina Central University, Durham, NC, United States; ^8^ Department of Pharmaceutical Sciences, College of Health and Sciences, North Carolina Central University, Durham, NC, United States

**Keywords:** micro-and nanoplastics, developmental neurotoxicity, metabolism, zebrafish, organoid

## Abstract

Micro- and nanoplastics (MNPs) are widespread environmental pollutants that pose significant health risks. They originate from industrial processes, consumer products, and environmental degradation, inducing oxidative stress through cellular dysfunctions such as membrane interaction, internalization, mitochondrial damage, inflammation, metal ion leaching, and impaired antioxidant defense. Despite increasing evidence of their toxicity—particularly developmental neurotoxicity (DNT) and mitochondrial impairment—our understanding remains limited due to the high costs of animal studies, which reduce the overall size of experimental data. This underscores the urgent need for alternative test methods that are cost-effective, rapid, and translational. This review examines new approach methodologies (NAMs) for DNT assessment, addressing the ethical, financial, and translational limitations of animal models. NAMs integrate three complementary non-animal models that enhance conventional testing. First, zebrafish models provide organismal insights into behavioral and neurodevelopmental outcomes at minimal cost. Second, neuronal organoids replicate human-specific neurodevelopmental processes in a 3D system, offering mechanistic insights. Lastly, human cell lines enable high-throughput screening, integrating findings from zebrafish and organoid studies. Establishing a new paradigm for DNT testing is crucial for faster and more efficient toxicity and risk assessments, ultimately protecting public health. Standardizing and gaining regulatory acceptance for NAMs will improve predictive accuracy and broaden their application in environmental toxicology. Advancing these methodologies is essential to addressing the risks of MNP exposure while promoting ethical and sustainable research practices.

## 1 Introduction

Micro- and nanoplastics (MNPs) have emerged as a global public health concern. These small plastic particles, ranging from microplastics (MPs, ≤5 mm) to nanoplastics (NPs, ≤1 µm), are predominantly derived from petroleum-based polymers. They originate from various sources, including the degradation of larger plastic debris, industrial emissions, and consumer products such as cosmetics and textiles (Barboza et al., 2018). MNPs infiltrate aquatic and terrestrial ecosystems, exposing human tissues and organs through ingestion, inhalation, and dermal contact (Horvatits et al., 2022; Hong et al., 2023; Campen et al., 2024; Saha and Saha, 2024; Wang et al., 2024). Their unique physicochemical properties enable them to leach into the environment, carry toxicants on their surfaces, cross biological membrane barriers, and disrupt cellular processes (Li et al., 2024). These findings highlight MNPs contamination as a growing health risk and underscores the urgent need to study MNPs health effects to aid in risk assessment and management.

Recent studies indicate MNPs can impair metabolism, immune function, and neuronal systems while exerting direct toxic effects on mitochondria, redox mechanisms, and signaling pathways (Prust et al., 2020; Yee et al., 2021; Yang et al., 2022; Ali et al., 2024). It was also noted that early-life exposure during critical brain development stages has been linked to neurotoxic effects, including neurotransmitter dysregulation, potentially leading to long-term cognitive and behavioral deficits (Prust et al., 2020).

Rodent models have been the standard for DNT testing but face ethical, logistical, and translational challenges, prompting efforts to reduce animal use (Bal-Price et al., 2010). Additionally, rodent studies are costly and labor intensive, limiting their scalability for testing emerging toxicants like MNPs (Du et al., 2024). Furthermore, physiological and developmental differences between rodents and humans often make it difficult to translate the results to human health (Krewski et al., 2010).

To overcome these limitations, alternative models that are high throughput, scalable and better adapted to human biology, including metabolic processes, are needed. Advances in NAMs provide ethical and predictive tools for DNT testing. NAMs provide critical insights into the developmental neurotoxicity of MNPs without relying on *in vivo* models and enable more sustainable and human-relevant research practices.

This review highlights how NAMs can transform current practices and promote a shift towards sustainable and accurate methods for assessing MNP-related global health risks.

## 2 Mitochondria is a potential target for MNPs

Mitochondria is vital organelles involved in energy metabolism, ATP production, fatty acid oxidation, calcium homeostasis, macromolecule biosynthesis, and cellular signaling pathways (Casanova et al., 2023; Popov, 2023). Mitochondria are susceptible to damage from chemicals, nanomaterials, and environmental pollutants resulting in dysfunction (Lee et al., 2022; Yao et al., 2024). Mechanisms of mitochondrial toxicity include reduced membrane potential, impaired enzymatic activity, disrupted ATP production, calcium dysregulation, and pro-apoptotic factor release (Tait and Green, 2012; Zong et al., 2024). Exposure to MNPs has been linked to mitochondrial dysfunction, such as impaired membrane potential, reduced respiratory complex activity, disrupted ATP production, increased reactive oxygen species (ROS), and oxidative stress, leading to inflammation, mitophagy, and apoptosis (Hu and Palic, 2020; Das, 2023). These effects contribute to various health issues, including metabolic and developmental disorders (Ali et al., 2024; Jeong et al., 2024). However, *in vivo* evidence of mitochondrial accumulation of MNPs remains limited (Silva et al., 2023), and further research is necessary to elucidate size- and type-dependent MNP-induced mitochondrial toxicity and its implications for developmental neurotoxicity.

MPs commonly found in our environment are primarily composed of polyethylene, polystyrene, polyvinyl chloride, polypropylene, among others (Gan et al., 2023). In rodent models, polyethylene terephthalate MPs reduced mitochondrial membrane potential, increased Bax protein expression, and decreased PARP, caspase-3, and Bcl-2 expression in H9C2 cells (Lu et al., 2024). MNPs exposure elevated ROS levels, leading to mitochondrial damage, reduced antioxidant enzyme activity (SOD, CAT, GSH-Px), disrupted redox balance, and cardiomyocyte apoptosis (Lu et al., 2024). Studies in zebrafish showed that chronic exposure to PP MNPs caused oxidative stress in the brain and liver, reducing mitochondrial respiratory efficiency and depolarizing mitochondrial membranes (Felix et al., 2023; Liu et al., 2024). These disruptions impaired respiratory complexes (e.g., Complex II, IV), increased ROS production, and triggered inflammatory and apoptotic pathways (Felix et al., 2023). In SH-SY5Y human cell lines, MNP exposure similarly caused increased ROS, mitochondrial damage, and apoptosis, disrupting cellular energy metabolism (Prust et al., 2020).

Despite progress, a standardized classification system for MPs based on size, shape, composition, and charge is unavailable. While organ-level MNP effects are studied, research on their developmental toxicity, especially DNT, is limited, highlighting the need for advanced models.

## 3 Zebrafish as alternative model for DNT testing

### 3.1 Overview of zebrafish as a NAM in toxicology

Zebrafish’s transparency, external fertilization, and rapid development make it ideal for high-throughput, real-time toxicology studies. With over 70% genetic homology to humans, including conserved neural pathways, they have played a critical role for neurotoxicity research (Kalueff et al., 2014). Historically used in environmental toxicology, the use of zebrafish is favored in DNT studies. Their small size, high fecundity, and low cost makes them ideal for fast turnover and scale-up chemical screening, such as MNPs (Planchart et al., 2016). Zebrafish thus emerge as an ethical, efficient, and human-relevant model in advancing toxicological research.

### 3.2 Zebrafish in MNP exposure studies

Zebrafish have recently emerged as a valuable model for assessing the developmental effects of MNPs. Research indicates that MNP exposure disrupts early neurodevelopment by inducing oxidative stress (Felix et al., 2023; Liu et al., 2024), apoptosis (Liu et al., 2024), and altering neurotransmitter balance (Prust et al., 2020). MNPs can also induce behavioral changes, such as altered locomotion, serve as sensitive indicators of neurotoxicity (Malhotra et al., 2019; Wu et al., 2024). For example, environmentally relevant MNP concentrations in zebrafish embryos disrupted motor activity and reduced neuronal connectivity, highlighting their potential to impair central nervous system (CNS) development (Barboza et al., 2018). Biomarkers like oxidative stress and apoptotic signaling further clarify the pathways leading to MNP toxicity.

Zebrafish model is also used to explore the size- and type-dependent effects of MNPs. Exposure to polystyrene nanoplastics (20 nm, 0.3–9 mg/L) caused morphological deformities, reduced body length, lower survival and hatching rates, impaired locomotion, and inhibited motor neuron development. These effects were associated with an increase in ROS, decreased superoxide dismutase (SOD) activity, and upregulation of genes related to apoptosis (e.g., *bcl2a* and *caspase-3a*). Additionally, nanoplastics altered the expression of neurodevelopmental genes (*gli2a, neurog1, syn2a*), underscoring ROS-induced apoptosis as a critical mechanism (Cao et al., 2024).

MNPs, particularly MPs, can increase their toxicity potential through adsorption by pollutants such as PCBs, PAHs, pesticides, and heavy metals). These contaminants adhere to MPs' large surface area and their hydrophobic character induces higher concentration in aquatic organisms (Gupta et al., 2024). For example, during zebrafish embryogenesis, polystyrene microplastics (PS-MPs) and perfluorooctane sulfonic acid (PFOS) exhibit synergistic toxicity. PS-MPs adsorb PFOS, cummulating oxidative stress, disrupting antioxidant enzyme activity, and impairing neurotransmission through reduced acetylcholinesterase (ACHE) activity and elevated acetylcholine (ACh) levels. Co-exposure also causes morphological defects, such as pericardial edema and altered yolk sac area, emphasizing the amplified metabolic disruptions initiated by contaminant adsorption (Gupta et al., 2024).

### 3.3 Challenges and future directions

Zebrafish models offer advantages for DNT testing but face challenges, including replicating chronic low-dose exposures and quantifying the extent of MNP bioaccumulation in embryos (Lei et al., 2023). More research is needed on the multigenerational effects of MNPs on neurodevelopment and behavior. Standardized protocols could improve reproducibility and regulatory acceptance, solidifying zebrafish as a key model in neurotoxicity testing. In addition, the use of zebrafish embryos for developmental toxicity assays was well established. It allows multiple endpoints evaluation related to lethal or sub-lethal effects along with developmental defects (Ducharme et al., 2013; Jin et al., 2013; Shen et al., 2020; Elersek et al., 2021). Zebrafish embryos also assess MNP toxicity through lethality, inflammation, oxidative stress, apoptosis, neurodevelopment, and behavior assays, highlighting environmental and health risks.

## 4 Neural organoids as human-relative NAM for DNT

### 4.1 The emergence of neural organoids in toxicology

A neural organoid is a 3D model that captures key aspects of brain development. This biological model includes an appropriate cellular diversity, structural organization, and maturation that make it a valid tool for studying the neurodevelopment and neurotoxicity (Lancaster and Knoblich, 2014). 3D neural organoids are derived from pluripotent stem cells (PSCs) and have transformed the study of DNT (Wu et al., 2025). Comparing to conventional 2D cell cultures, neural organoids mimic human brain development by providing a 3D structure that supports complex cellular interactions and regional organization of the brain. This unique capability makes organoids suitable for investigating brain region-specific effects of environmental toxicants, such as MNPs (Lancaster and Knoblich, 2014).

The emergence of neural organoids as research tools has filled critical gaps in translational studies by replicating essential neurodevelopmental processes, including neurogenesis, synaptogenesis, and circuit formation. Neural organoids provide human-specific insights, overcoming rodent models' limitations in replicating human neural development. This linking of *in vitro* assays and *in vivo* systems enhances our ability to investigate the developmental impacts of toxicants like MNPs, providing a more human relevant and ethical approach to studying human DNT (Quadrato et al., 2016).

### 4.2 Application of neural organoids in MNP neurotoxicity studies

Recent advances have underscored the utility of neural organoids in assessing the neurotoxicity of MNPs including neuroinflammation, disruption of neurogenesis, and alteration of neural connectivity. RNA sequencing has identified molecular signaling pathways impacted by MNPs exposure, including oxidative stress and inflammatory signaling (Chen et al., 2023). For example, Tao et al. (2024) demonstrated that polystyrene nanoparticle exposure reduced neural progenitor cell proliferation and disrupted cell differentiation in neural organoids (Tao et al., 2024). Advanced techniques including calcium imaging and electrophysiological recordings have further highlighted functional deficits in organoids, revealing the developmental risks posed by MNPs during critical periods of brain growth (Yan et al., 2024).

Neural organoids provide a human-relevant model for studying MNP-induced mitochondrial dysfunction in developmental neurotoxicity. MNPs cause dose-dependent mitochondrial damage in brain organoids, with low doses causing mitochondrial swelling, cristae disruption, and vacuole formation and high doses resulted in severe mitochondrial vacuolation, membrane disappearance, and structural breakdown (Chen et al., 2023). These mitochondrial impairments were accompanied by broader cellular dysfunction, including membrane breaches and intracellular lipid droplet formation, highlighting mitochondria as the central target of MNP-induced developmental neurotoxicity (Chen et al., 2023).

Organoids also enable brain region-specific developmental toxicity studies. PSC-derived cortical organoids exposed to PS-MPs (1 μm, endocytosed; 10 μm, affecting adhesion) at 5–100 μg/mL showed increased ROS, oxidative stress, and redox imbalance. Short-term exposure upregulated SOD2, while long-term exposure depleted antioxidants. Mitochondrial dysfunction impaired cortical differentiation, cell viability, and neural marker expression (Hua et al., 2022). These effects highlight oxidative stress, mitochondrial impairment, and depletion of the antioxidant response as central mechanisms of MNPs-induced neurotoxicity and effects on brain region development.

These studies confirm the relevance of neural organoids in understanding MNPs-induced neurotoxicity and promote sustainable, human-relevant research methods for assessing neural developmental risks.

### 4.3 Challenges and future directions

Neural organoids hold great promise but face challenges due to a lack of vascularization and immune components compared to *in vivo* conditions (Quadrato et al., 2016). Recent advances such as vascularizing neural organoids via co-culture with vascular cells, vascular organoid integration, and stem cell co-differentiation, are addressing the issue (Nwokoye and Abilez, 2024). Advancements in Organ-on-a-chip now integrate perfusable vasculature into organoids, enhancing physiological modeling *in vitro* (Li et al., 2024; Zhang et al., 2024). These approaches aim to overcome the constraints on tissue size and achieve in vivo-like functionality by providing essential nutrients and oxygen to the neural organoid core (LaMontagne et al., 2022).

## 5 Human cell lines as an *in vitro* NAM for DNT screening

### 5.1 Utility of human cell lines in DNT testing

Human cell lines are important tools in DNT testing and they offer ethical and reproducible alternatives to animal models. Neuronal lines such as SH-SY5Y and IMR-32, derived from human neuroblastoma, are commonly used to study neuronal differentiation, signaling pathways, and DNT toxicant responses (Xicoy et al., 2017). Complementary non-neural lines, such as Caco-2, derived from intestinal epithelial cells, help to study the uptake and systemic distribution of toxins. These cell lines are invaluable in their simplicity, reproducibility, and mechanistic insights. They facilitate the research on key processes in toxicant-induced developmental neurotoxicity, including oxidative stress, apoptosis, and mitochondrial dysfunction.

### 5.2 Key findings from human cell line studies on MNP toxicity

Research on MNPs has consistently shown cytotoxicity, oxidative stress, mitochondrial dysfunction, and inflammation in human cell lines. In SH-SY5Y cells, MNP exposure induces oxidative stress, ROS accumulation, and structural damage of mitochondria and dysfunction, which disrupt energy metabolism and induce apoptosis (Prust et al., 2020). Dose-response studies show that smaller NPs exhibit greater cytotoxicity due to their increased surface area and cellular uptake (Ruan et al., 2023; Mahmud et al., 2024). In addition, MNPs exposure upregulates inflammatory cytokines such as IL-6 and TNF-α, highlighting their potential to induce inflammation (Donisi et al., 2024; Martin-Folgar et al., 2024). All these findings underscore the importance of human cell lines in evaluating MNPs DNT toxicity. The comparative summary is shown in the Table 1.

**TABLE 1:** Comparative summary of zebrafish, neural organoids, and human cell lines in DNT testing.

Model	Strengths	Limitations	Applications
Zebrafish	Cost-effective, high-throughput, and capable of modeling organismal responses; transparent embryos	Limited human relevance in complex neurodevelopment; difficulties in modeling chronic exposure.	Behavior analysis, neurodevelopmental studies, validation of findings from *in vitro* systems.
Neural Organoids	3D architecture mimics human-specific brain development, suitable for mechanistic studies.	High variability in culture methods; lack of vascularization and representation of the immune system.	Mechanistic insights, regional neurodevelopmental analysis, and omics-based evaluations.
Human Cell Lines	Easy to culture; reproducible; high-throughput capacity; ideal for molecular and biochemical studies.	Limited ability to mimic *in vivo* complexity and systemic interactions.	Initial screening for cytotoxicity, oxidative stress, and dose-response relationships.

### 5.3 Challenges and future directions

Human cell lines lack the complexity and 3D structure of tissues, so they cannot replicate the cellular interactions in the body. Important systemic interactions, such as those between cells, immune responses, or the dynamics of the blood-brain barrier, are critical for understanding the overall effects of toxicants but cannot be modeled effectively with cell lines alone. These limitations highlight the need for incorporating cell lines into more complex models, such as organoids or *in silico* simulations. Co-culture systems and microfluidic “organ-on-a-chip” technologies offer complementary solutions, creating more physiologically relevant environments to bridge these gaps.

## 6 An integrated NAM approach for developmental neurotoxicity testing

A tiered, integrated testing battery combining human cells, neural organoids, and zebrafish models can improve the efficiency and relevance of DNT studies while reducing the use of animals. In this approach, human cell lines such as SH-SY5Y serve as an initial screening platform for the identification of potential neurotoxicants and provide scalable and cost-effective assays for the detection of early molecular signaling. Next, neural organoids enable deeper investigation of region-specific neurodevelopmental toxicity in the 3D human *in vitro* model, enabling more comprehensive dose-response analysis and pathway discovery. Finally, zebrafish provide an organismal perspective by assessing systemic and behavioral effects, including locomotion and memory, capturing complex outcomes and providing information on long-term effects. This stepwise strategy is consistent with the 3Rs—replacement, reduction, and refinement—by focusing animal-based approaches on the final confirmation stages. Regulatory acceptance of NAMs depends on robust validation, standardization, and ongoing collaboration, as demonstrated by programs such as US Tox21 and the EU Joint Research Center. These efforts lead to shared performance criteria, foster reproducibility, and support the incorporation of omics technologies and machine learning, which refine biomarker detection and improve predictive power. By addressing technical variability and promoting confidence in NAMs, such collaborative efforts accelerate their formalization, ensuring broader acceptance in the regulatory framework.

## 7 Conclusion and future perspectives

### 7.1 Conclusion

NAMs are revolutionizing DNT testing by offering human-relevant, scalable, and high-throughput solutions. These methodologies have shown significant potential to reduce reliance on traditional rodent animal models while providing valuable mechanistic insights into the developmental neurotoxic effects of environmental contaminants, including MNPs (Krewski et al., 2010).

The development of *in vitro* cell-based assays and the integration of new approach methodologies for developmental neurotoxicity screening and risk assessments can be traced back to 2008, when the Tox21 Consortium was established among OECD countries and federal agencies in the U.S. (Tice et al., 2013; OECD, 2023). Recent advances have highlighted the different strengths of zebrafish, neural organoids, and human cell lines in DNT research. Zebrafish models provide cost-effective, organismal insights into the behavior and neurodevelopmental outcomes (Bailey et al., 2013). Neural organoids bridge the gap between *in vitro* 2D cell models and *in vivo* testing systems, enabling the study of complex cellular interactions and region-specific neurodevelopment (Lancaster and Knoblich, 2014). Human cell lines are efficient for high-throughput screening and mechanistic studies, revealing cellular responses like oxidative stress, inflammation, and apoptosis (Xicoy et al., 2017).

Collectively, these NAMs establish a robust and adaptable framework to address the challenges of DNT testing in an evolving regulatory landscape, enhancing our understanding of neurotoxicity while reducing animal use. Ongoing efforts to integrate available NAMs into toxicity assessments—led by multiple federal agencies, including the U.S. EPA, NCATS, NIEHS, and FDA—are expected to further accelerate the identification of optimal assays for future toxicology studies.

### 7.2 Future perspectives

NAMs still face challenges in DNT testing that necessitate standardization and validation for acceptable reproducibility and for gaining regulatory acceptance. OECD zebrafish guidelines and neural organoid harmonization are efforts in this direction, but more steps need to be taken before NAMs can be embraced by the scientific community as reliable alternative models (Sobanska et al., 2018). Recent studies emphasize acute and high doses MNPs exposure, leaving a gap in understanding MNPs' cumulative effects on neural development. Future research should also focus on long-term, low-dose MNPs exposure, mimicking human relevant inhalation or dietary intake level (Prust et al., 2020). Meanwhile, integrating omics and machine learning with NAMs increases predictability and helps identify novel DNT biomarkers. (Zhou et al., 2024). In addition, fostering collaboration between researchers working on standardized MNPs in research, industry stakeholders and regulators is critical to accelerating the use of NAMs. By addressing these challenges and taking advantage of these opportunities, we can advance DNT testing using NAMs, contributing to more ethical, human-relevant toxicology research.
